# No bed for a dying child: the shortage of paediatric intensive care in Pakistan

**DOI:** 10.7189/jogh.16.03003

**Published:** 2026-02-06

**Authors:** Aqsa Elle, Muhammad Hamza Shafiq

**Affiliations:** 1 Dow University of Health Sciences, Karachi, Pakistan; 2 Bolan Medical College, Quetta, Pakistan

## Abstract

Pakistan’s neonatal and under-five mortality rates remain among the highest in South Asia. This challenge is exacerbated by a critical shortage of paediatric intensive care staff, with <30 trained specialists serving >80 million children. Recent national data indicate that only 70% of accredited paediatric hospitals have functional paediatric intensive care units, and nearly half lack adequate nurse-to-patient ratios. These shortages are most severe in rural regions, where delayed access to life-saving interventions contributes to preventable deaths. Emerging solutions such as telemedicine, neonatal care units within general hospitals, and public-private partnerships demonstrate potential for scalable reform. Strengthening paediatric critical care is essential to achieving Sustainable Development Goal 3.2 and ending preventable child deaths by 2030.

During a recent on-call shift in the ophthalmology department, Dr Elle witnessed a tragedy that underscores Pakistan’s severe shortage of paediatric intensive care. A seven-year-old boy, injured in a road traffic accident, arrived in critical condition. His mother and sister stood by helplessly as they were told no paediatric intensive care unit (PICU) bed was available. Although the surgical team confirmed that he required intensive care, the PICU declined admission, citing a lack of space and perceiving his survival chances to be slim. As his condition deteriorated in a general ward without adequate support, I felt the devastating weight of a health system unable to offer even the chance of survival.

Neonatal mortality stands at 41 per 1000 live births, and as of July 2023, the under-five mortality rate is 67 per 1000 [1]. This is not an isolated incident, but rather one that reflects a systemic crisis in paediatric critical care across Pakistan. The country has <30 trained paediatric intensivists serving >80 million children [2]. Most PICUs are concentrated in Karachi, Lahore, and Rawalpindi, leaving rural populations with limited access. Delays in essential interventions, such as mechanical ventilation, fluid resuscitation, and inotropic support, contribute to preventable deaths, especially among neonates. In many district hospitals, the absence of basic monitoring tools forces clinicians to rely solely on clinical judgment, often with devastating consequences.

Beyond infrastructure gaps, socioeconomic disparities further delay care. Families frequently travel long distances seeking treatment, often reaching tertiary facilities when a child’s condition has already progressed beyond recovery. High out-of-pocket costs at private hospitals push disadvantaged families toward overcrowded public facilities with limited capacity. These intersecting barriers contribute to a cycle in which preventable paediatric deaths become normalised.

## CURRENT STATUS OF PAEDIATRIC INTENSIVE CARE IN PAKISTAN

Recent national data reveal substantial deficiencies in paediatric critical care infrastructure. A survey using the ‘Partners in Health 4S’ (space, staff, stuff, systems) framework across 114 accredited paediatric training hospitals found that only 53 hospitals (70%) had a functional PICU, with 667 specialised beds, 217 mechanical ventilators, and only 20 trained intensivists across 16 units (30%). Nearly (47%) of PICUs had a nurse-to-patient ratio <1:3, highlighting severe workforce constraints. Compared to public facilities, private hospitals scored significantly higher on infrastructure and resource availability (*P* = 0.003) [3].

These findings reflect a critically underdeveloped system. Many public PICUs struggle with unreliable oxygen supply, equipment breakdowns, and shortages of essential medications. Additionally, Pakistan lacks a standardised national training pathway for paediatric critical care, leaving many units staffed by general paediatricians with limited exposure to intensive care management. The absence of structured training results in inconsistent quality of care and wide variations in outcomes across facilities.

## REGIONAL AND GLOBAL PERSPECTIVE

Compared with neighbouring lower-middle-income countries, Pakistan continues to lag in paediatric critical care investment. India and Sri Lanka have established fellowship programmes in paediatric intensive care and maintain national registries of critical care outcomes, enabling systematic quality improvement. Bangladesh’s expansion of district-level neonatal units, through collaboration with non-governmental organisations, has significantly reduced neonatal mortality in underserved regions. These examples demonstrate how coordinated national programmes can strengthen survival outcomes in resource-limited settings.

Countries such as Vietnam, Nepal, and Rwanda have achieved major reductions in child mortality through integrated referral systems, standardised triage protocols, and strong linkages between community and hospital care. These reforms dramatically reduced delays in the recognition and management of critical illness. Pakistan’s progress toward Sustainable Development Goal 3.2 will depend on similar system-wide alignment. Without strengthening emergency transport, referral pathways, and inter-hospital coordination, expanding PICU capacity alone will not substantially reduce mortality.

## APPROACHES TO ADDRESS THE SHORTAGES

Neonatal care units within general hospitals can expand intensive care capacity beyond major cities. Equipping these units with essential technologies, such as incubators, continuous positive airway pressure devices, and ventilators, and training local staff has successfully reduced neonatal mortality in comparable contexts [4]. Pakistan’s Lady Health Worker programme could be integrated into neonatal follow-up pathways to support early detection of complications.

Public-private partnerships can mobilise resources to build and operate PICUs. Successful public-private partnerships in Lahore have improved neonatal survival outcomes [5]. Expanding similar models to rural districts could reduce geographic inequities by ensuring that at least one functional PICU is available in each division.

Capacity-building programmes for paediatricians and nurses are essential. Structured training, simulation-based learning, certification programmes, and mandatory continuing education can elevate care quality in existing units. A national fellowship in paediatric critical care with rotations across multiple provinces would help establish a sustainable pipeline of trained specialists.

Several strategies can help bridge current gaps in paediatric critical care. Telemedicine platforms can connect rural health facilities with specialised PICUs, enabling real-time consultations. Remote monitoring of high-risk infants in low-resource settings has been shown to improve neonatal outcomes [6], and tele-intensive care units models in other lower-middle-income countries have reduced unnecessary transfers and mortality.

## FUTURE DIRECTIONS AND POLICY RECOMMENDATIONS

Strengthening paediatric critical care in Pakistan requires a coordinated, evidence-based strategy. Expanding PICU availability, integrating telemedicine, and investing in paediatric critical care training are immediate priorities. Low-cost innovations such as portable ventilators, solar-powered incubators, and locally manufactured monitoring systems can overcome infrastructure constraints.

Public-private partnerships can enhance resource sharing and sustainability, while a national paediatric critical care registry would facilitate data-driven decision-making and equitable resource allocation. Improving ambulance networks, standardising referral criteria, and establishing provincial command centres for real-time PICU bed management can significantly reduce treatment delays. Aligning these initiatives with national child health programmes and global commitments will be essential for measurable progress.

## CONCLUSIONS

The shortage of PICUs in Pakistan is both a moral and systemic challenge. Every preventable child death reflects gaps in access, training, and policy attention. By expanding PICU capacity, strengthening the healthcare workforce, leveraging telemedicine, and improving data systems, Pakistan can make meaningful progress toward Sustainable Development Goal 3.2 and ensure that no child is denied care due to a lack of a bed.
